# High temporal resolution of leaf area data improves empirical estimation of grain yield

**DOI:** 10.1038/s41598-019-51715-7

**Published:** 2019-10-31

**Authors:** François Waldner, Heidi Horan, Yang Chen, Zvi Hochman

**Affiliations:** 1 CSIRO Agriculture & Food, 306 Carmody Road, St Lucia, Queensland 4067 Australia; 2 CSIRO Data61, Underwood Avenue, Goods Shed North, 34 Village St, Victoria 3008 Australia

**Keywords:** Environmental sciences, Plant sciences

## Abstract

Empirical yield estimation from satellite data has long lacked suitable combinations of spatial and temporal resolutions. Consequently, the selection of metrics, *i*.*e*., temporal descriptors that predict grain yield, has likely been driven by practicality and data availability rather than by systematic targetting of critically sensitive periods as suggested by knowledge of crop physiology. The current trend towards hyper-temporal data raises two questions: How does temporality affect the accuracy of empirical models? Which metrics achieve optimal performance? We followed an *in silico* approach based on crop modelling which can generate any observation frequency, explore a range of growing conditions and reduce the cost of measuring yields *in situ*. We simulated wheat crops across Australia and regressed six types of metrics derived from the resulting time series of Leaf Area Index (LAI) against wheat yields. Empirical models using advanced LAI metrics achieved national relevance and, contrary to simple metrics, did not benefit from the addition of weather information. This suggests that they already integrate most climatic effects on yield. Simple metrics remained the best choice when LAI data are sparse. As we progress into a data-rich era, our results support a shift towards metrics that truly harness the temporal dimension of LAI data.

## Introduction

Estimating crop production, particularly that subject to international trade, is becoming an urgent imperative for global food security due to the growing world population, shifts in diets, and the development of biofuels. Instead of quantifying production directly, it is common practice to address its constituent terms: crop area and crop yield. The latter can itself be thought of as the product of genetic, environment, and management (G × E × M) factors. Agrometeorological models have long been deployed with some success to estimate yields on a regional basis, either based on statistical relationships relating yield to meteorological data or based on crop growth models that not only relate weather parameters to yield but also explain plant growth^1–3^. Satellite remote sensing has gradually become instrumental in assessing crop yields because several Vegetation Indices (VIs) derived from spectral data integrate some G × E × M effects, from meteorological factors such as precipitation and solar radiation to cropping practices such as fertilisation and irrigation. In fact, there is a considerable amount of within-field variance that is not explainable by meteorological data but that can be accounted for by multi-spectral satellite data^4^.

Numerous methods have been devised to predict crop yield based on satellite data and include for instance assimilation of satellite data into crop growth or light-use efficiency models^5,6^. Another straightforward approach to estimate crop yields is to establish an empirical relationship between ground-based yields and VIs or metrics describing VI time series. These empirical models rely on the correlation between spectral bands (and their combinations) and biophysical properties of the crops, such as Leaf Area Index (LAI), which are themselves related to final yields^7^. A large range of VIs has been tested, with mixed results, in different regions and for different crops including the well-known Normalised Difference Vegetation Index^8–11^. Capitalising on the ability to retrieve biophysical variables from satellite data^12,13^, some attempts have empirically correlate biophysical variables to yield^14,15^. The principle remains the same for biophysical variables as for VIs: metrics are first extracted from time series of biophysical data and are then related to measured yields.

The lack of sufficient field- or pixel-level yield measurements for model calibration and validation has long hindered attempts to deploy empirical models at scale for operational monitoring. In addition, empirical models are specific to the crop cultivars, the crop growth stages, and the geographical regions they are calibrated on^16,17^. Therefore, they do not generalise well in data-poor contexts. An elegant solution, referred to as the scalable satellite-based crop yield mapper or SCYM, was recently proposed to solve the lack of availability of calibration data^18^. In essence, SCYM calibrates empirical yield models with modelled data obtained from the Agricultural Production System sIMulator^19^ (APSIM), a thoroughly-validated crop model, rather than with *in situ* yield measurements. The role of the crop model is to generate a large number of simulations that span a realistic range of soil, climate, and management conditions in the region of interest so that robust statistical relationships may be established between yield and crop canopy descriptors. SCYM models can then be transferred and applied to satellite images to map yields across vast areas. It has been tested for multiple crops and countries and explained, for instance, half of the wheat yield variability in India^20^. While several avenues still exist for improving the accuracy of SCYM models, its strategy of using simulations from regionally-tuned and parameterised crop models to calibrate empirical models in lieu of costly *in situ* yield measurements paved the way to deploying empirical yield models anywhere in the world.

Empirical yield estimation from space has also been constrained by the trade-off between the spatial and temporal resolution which restricted the use of high spatial and temporal images for agricultural applications^21^. Data availability hampered multi-temporal analyses or these were limited to time series with coarser spatial resolutions, leading to pixel purity issues^22^, which are particularly challenging in complex landscapes^23^. Consequently, it is likely that the choice of metrics, *i*.*e*., time series descriptors used to predict grain yield, was driven by practicality and data availability rather than by systematic targetting of critically sensitive periods suggested by knowledge of crop physiology. Constellations of satellites, *e*.*g*., Sentinel-2 A and B^24^ (5-day revisit, 10-m resolution), or the Dove constellation from Planet^25^ (daily global coverage at 3 m with 175+ satellites), have opened an avenue for overcoming these spatiotemporal restrictions. Therefore the advent of hyper-temporal data offers an unprecedented opportunity to revisit empirical yield estimation and explore new alternatives to exploit finer and denser temporal patterns.

Our overarching goal is to advance routine yield assessment across the Australian grain zone based on satellite observations and to do so by leveraging the capabilities of the most recent and upcoming imaging systems. Here we evaluated how the density of LAI observations affects performance and the choice of metric required to achieve optimal performance. We premised our work on three observations widely supported by evidence from the literature: (1) crop growth models can accurately simulate plant growth, yield, and leaf area. For instance, the wheat model within APSIM has been extensively validated across Australia and internationally in a range of experimental and farm conditions^26–33^; (2) leaf area index can be retrieved from satellite images^13,34^; (3) empirical yield models calibrated off simulated yields, *i*.*e*., *a la* SCYM^18,20^, provide reasonable spatially-explicit yield estimates when applied to remotely-sensed data. The direct implication is that data generated by crop models can be used to evaluate *in silico* different forms of empirical models.

The *in silico* approach has the following advantages: (1) the range G × E × M conditions that can be explored *in silico* is larger than what observational data would otherwise allow, which improves generalisation; (2) *in silico* data can simulate forthcoming temporal resolutions or mimic current imaging systems lacking sufficient archive data, which facilitates systematic comparisons; and (3) *in silico* testing provides these results for a fraction of otherwise prohibitive costs associated with image acquisition and *in situ* yield data. Here, We deployed APSIM to simulate wheat growth during 30 consecutive seasons under 10 management scenarios at 50 locations across Australia. These simulations provided time series of LAI and yields which were used to calibrate and evaluate different types and configurations of empirical models. It should be emphasised that our objective was neither to predict past wheat yields nor to apply our findings to remotely-sensed data. Rather we sought to generate likely LAI time series and yields under a variety of growing conditions and evaluate the stregnth of their relationship under different scenarios represnetating present and forthcoming observation capabilities.

Our main contributions are three-fold:

- We provide a systematic comparison of LAI metrics and highlight that linear empirical models with advanced metrics (*e*.*g*., Senescence Fit, or Fourier Decomposition) capture up to 80% of the yield variability. This is remarkable because the generalisation of empirical models has often been criticised. Therefore, this suggests that models using metrics that truly harness the temporal dimension of the LAI data can achieve regional to national relevance;
- We evaluate the contribution of weather variables to the overall performance and show that they can double the accuracy of models calibrated with simple metrics such as peak LAI. Average and cumulative maximum temperatures, as well as cumulative post-anthesis rainfall, are particularly strong predictors of grain yield. However, with advanced metrics, there is no significant improvement when adding weather variables because their effects are already captured by the metrics;
- We quantify the loss of accuracy that occurs when the temporal resolution decreases. In particular, we show that simple metrics remain competitive in data-poor contexts.

These results can serve as a guideline for selecting an appropriate metric depending on the temporal availability of earth observation data at hand.

## Results

### Accuracy of the prediction models without weather variables

**Figure 1:**
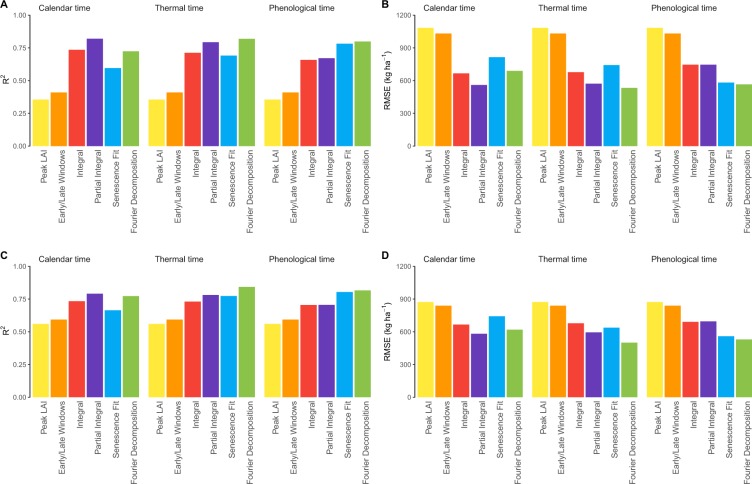
Performance indicators of the empirical models without weather variables (**A**,**B**) and with weather variables (**C**,**D**).

Wheat crops were simulated under nine management strategies for 15 years at 50 locations representative of the Australian grain zone from which we obtained daily LAI, thermal time, phenological stages, and the associated yields. Six types metrics were then extracted from the LAI time series (Peak LAI, Early/Late Windows, Integral, Partial Integral, Senescence Fit, and Fourier Decomposition) for three time scales (calendar, thermal and phenological time, the last two adjusting the time series for growth rate). The metrics were finally regressed against grain yields. We computed the R^2^ (Fig. 1A,C) and the RMSE (Fig. 1B,D) to evaluate the performance of the regression models with and without weather variables.

Between 30% and 78% of the yield variability can be explained by LAI features depending on the choice of features and time scale. Note that the Peak LAI and the Early/Late Windows approaches are by definition insensitive to a change in the time scale. Peak LAI consistently explained the least variance (R^2^ = 0.36; RMSE = 1,100 kg ha^−1^) followed closely by the two windows metrics (R^2^ = 0.41; RMSE = 1,032 kg ha^−1^). Using calendar time, the Partial Integral metric reached the highest coefficient of determination (R^2^ = 0.77; RMSE = 560 kg ha^−1^) followed by the Integral and the Fourier metrics. Interestingly, the ranking of the best performing methods changed with respect to the time scale. For instance, the Senescence Fit and the Fourier Decomposition metrics were both improved when accounting for thermal time or phenology: R^2^ values increased from 0.60 to 0.69 and 0.78 and from 0.72 to 0.80, respectively. It is worth noting that these two metrics are related to a smoothing of the time series. Switching to phenological and thermal times can lead to worse results than calendar time in some cases, *e*.*g*., Integral and Partial Integral. This drop might be partly attributed to the current calculation of thermal time in APSIM, to the LAI features themselves, and to the abrupt transitions inherent at some phenological stages.

### Contribution and importance of weather variables

The R^2^ of the linear model based only on the weather features reached 0.36 and the corresponding RMSE was 1,044 kg ha^−1^, which was slightly better than the accuracy reached by the peak LAI metric (R^2^ = 0.36; RMSE = 1,100 kg ha^−1^). Adding weather features was particularly beneficial to those models using simpler metrics but had little effect otherwise (Fig. 1C,D). They help reduce by half the difference in accuracy between the poorest and best models. For instance, the R^2^ of the peak LAI method reached 0.56 whereas the R^2^ of the Fourier approach only increased by 0.01.

**Figure 2:**
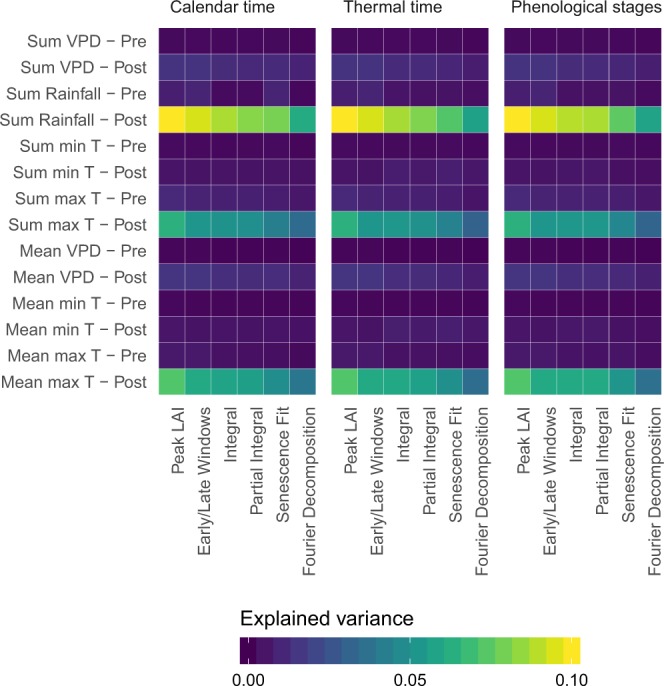
Contribution of weather variables to the R^2^. Rows represent the weather variables and columns correspond to models based on different metrics pre and post-anthesis.VPD: vapour pressure deficit; max T: minimum temperature; min T: minimum temperature.

We evaluated the contribution of the weather variables to the model R^2^ (Fig. 2). The weather variable with the highest contribution is the cumulative rainfall after the LAI peak. The sum and average maximum temperature post-peak were also important. The remaining variables only exhibited a very low contribution (<0.03) to the R^2^. The contribution of weather variables decreased as they were combined with LAI metrics derived from more advanced methods. Differences between the contribution of the variables computed for different temporal scales were small.

### Robustness to reduced temporal frequencies

**Figure 3:**
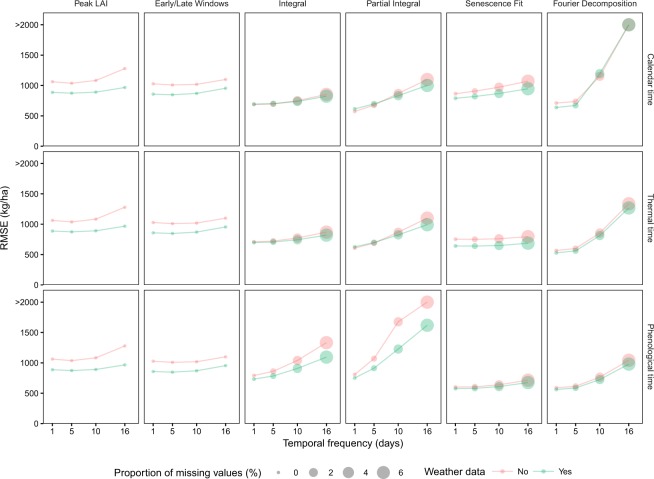
Average accuracy of the six metrics as a function of the temporal frequency of the input time series for the three time scales. The size of the point indicates the proportion of missing values resulting in failed LAI predictions due to a lack of input data.

Reducing the temporal frequency and accounting for cloud contamination reduced the prediction accuracy and increased the number of predictions with missing values (Fig. 3). However, this effect was metric-specific and three groups could be defined. The first group contained simple metrics (Peak LAI and Early/Late windows) that displayed robustness to a reduction of the temporal density, with little impact on the accuracy and the proportion of missing values. The second group (Integral and Senescence Fit) maintained a relatively stable accuracy but this was achieved at the expense of a higher rate of missing values. The third group (Partial Integral and Fourier Decomposition) was sensitive to a reduction of the temporal frequency both in terms of accuracy and failed predictions. This underscores that some metrics, in order to explain yield variations, require a higher temporal density, *i*.*e*., less signal contamination can be tolerated, while others are more robust and can be applied on sparser time series. This also varied with respect to the time scale: the Partial Integral approach was effectively best for calendar time whereas phenological time was best for the Fourier Decomposition and Senescence Fit approaches when the observation frequency was >5 days.

### Mapping optimal metrics

**Figure 4:**
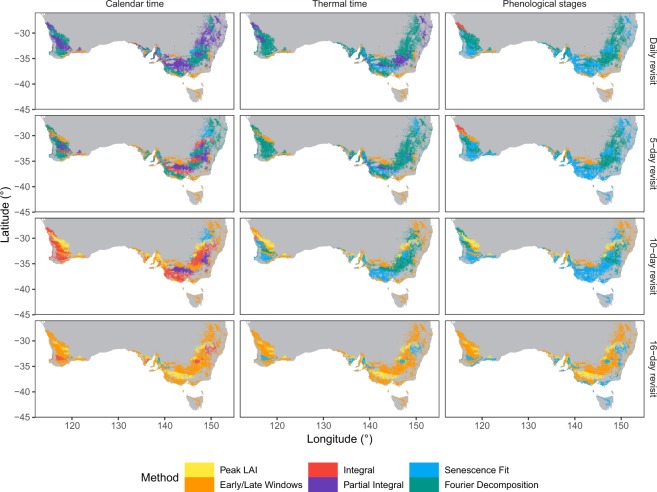
Most accurate approaches across the Australian wheat area as a function of temporal resolution and time scale.

Finally, we mapped the optimal metrics across the Australian wheat production area (Fig. 4). These varied by location and by time scale which was consistent with previous results: as the temporal frequency becomes sparser, the Peak LAI and the Early/Late season approaches became increasingly the preferred choice. This underscores that, while the Partial Integral, the Senescence Fit, and the Fourier Decomposition metrics yield higher accuracy, their use can only be recommended when the temporal resolution is ≤5 days.

## Discussion

There is a high demand for grain yield estimates for food security, logistics, or crop insurance purposes. Empirical models have been criticised for their lack of generalisation, *i*.*e*., their applicability has been found to be limited to specific crop cultivars, crop growth stages, and geographical regions^17,35^. The ever-increasing availability of satellite data is a potential boon for delivering accurate grain yield predictions across vast areas. To quantify the potential gains of leveraging hyper-temporal data, we developed an *in silico* approach which uses the crop growth model APSIM as data generator and calibrated empirical models with a series of LAI metrics for different time scales and temporal resolutions. The poor accuracy obtained with simple metrics suggests they cannot capture such diversity with single national-scale models and that locally-tuned models could improve their prediction skills^8^. Advanced metrics achieved high accuracies with single empirical models, which provides evidence that metrics harnessing the temporality of the data have national relevance.

**Figure 5:**
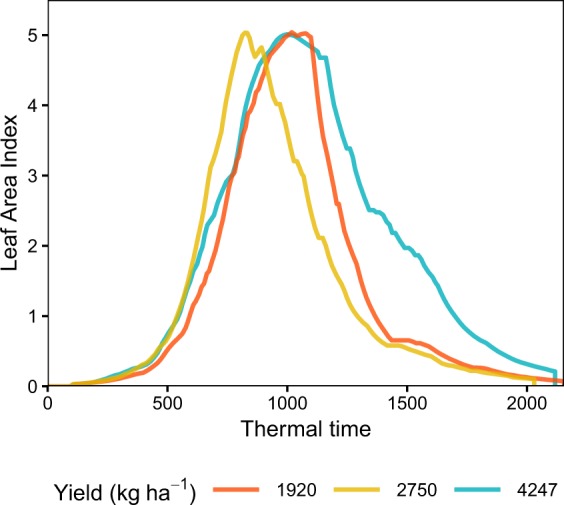
Illustration of the limitations related to the peak LAI approach. All time series have a similar peak LAI value but different shapes and timing of events, resulting in different yields.

Peak LAI consistently registered some of the worst predictions despite its widespread use in the remote-sensing literature. The strength of the peak LAI relationship to yield (R^2^ = 0.36) was weaker than what previously reported^9,36^, which could be partly explained by the larger range of G × E × M effects encountered in this study. Peak LAI completely disregards the critical period of grain filling^37^ and therefore cannot capture the impact of post-peak events such as terminal drought, which is often experienced in Australia^38^. Besides, large biomass early in the season does not necessarily result in large grain yield. These shortcomings are illustrated in Fig. 5, where three time series reach similar peak LAI values but end up with drastically different yields. Therefore, peak LAI is most useful to provide early estimates of grain yield. Integrating temporal profiles outperformed the peak LAI approach because the cumulative effect of photosynthetic apparatus efficiency during the entire growing period was taken into account. Conditions affecting the flag leaf and the penultimate leaf, which are the most active parts from a photosynthetic perspective, greatly influence final grain yield^39^.

An important contribution of this research is to better understand the optimum application conditions of different metrics depending on temporal resolution and availability of the LAI data. While advanced methods such as the Senescence Fit or the Fourier Decomposition outperformed simple methods when hyper-temporal data are at hand, the latter should be preferred with sparse time series. Not only do these simple methods perform better in data-poor contexts but they are also less sensitive to missing values. This confirms that biweekly composites cannot adequately characterise crop productivity if crop critical periods are smoothed by the compositing algorithm^8^ and implies that the use of maximum LAI or Early/Late Windows LAI was driven by practicality and data availability rather than by systematic targetting of critically sensitive periods suggested by crop physiology. Integrating radar data^40,41^ and blending lower resolution time series^42,43^ are two mitigation options to increase data frequency in areas with persistent cloud cover. Attention should be paid to correct the spatial scaling bias when fusing LAI data^44,45^ because it does not correlate linearly with spatial resolution^46,47^. Given the unprecedented revisit cycle of current Earth Observation systems, data fusion capabilities, and the prospects of future missions, our modelling suggests that the time is ripe for a shift towards the use of data-intensive metrics for empirical yield estimation.

Weather variables were instrumental in doubling the R^2^ of the models calibrated with simple metrics but had a marginal effect on those using advanced metrics. This suggests that half of the accuracy of scalable satellite-based crop yield mappers parametrised with the Early/Late Windows metric^18,48^ can be attributed to weather variables and confirms that the explicit consideration of weather was the main factor explaining the better performance of the original scalable satellite-based crop yield mapper compared to a peak VI model^20^. The three most important variables were cumulative rainfall, cumulative maximum temperature and average post-peak maximum temperature when the evaporative demand is higher. They all relate to water and heat/drought stresses and, by extension, to stored soil water which is critical for the growth of rainfed wheat in Australia. During grain filling, high temperature decreases leaf chlorophyll content and accelerates senescence^49^, leading to a shorter grain filling duration with an ultimate decrease in individual grain weight and yield that cannot be compensated by the higher grain filling rate under high temperatures^50^. The appropriate combination of predictors to include in empirical yield models depends on the cost of obtaining and using such data compared to the benefits^4^. The choice of adding weather predictors depends on their availability and on the temporal resolution of the LAI time series. This suggests that, if accurate and appropriate weather data are not readily available and if the temporal resolution allows advanced metrics to be robustly derived, the prediction model may be shrunk to LAI variables.

In advancing routine yield assessment, our study illustrates the importance of the temporal resolution for accurate yield prediction and provides some guidelines to inform on the choice metric depending on data availability. To some extent, the accuracy values reported here may represent the upper bound of what could be achieved when applying empirical models calibrated with crop model data to satellite imagery and, therefore, some considerations about the premises of this study ought to be raised. First, we assumed that yields and LAI could be accurately simulated by crop growth models. While their ability to predict grain yield has been thoroughly evaluated and confirmed, less emphasis was on modelling LAI, *e*.*g*., it has been reported that APSIM tends to slightly overestimate LAI^33^. Nonetheless, crop models provide water-limited yield potential (the yields that can be achieved when water and the environment are the only limiting factors) rather than actual yields (the yields achieved in commercial fields) so discrepancies are expected, *e*.*g*., where biotic stresses have a significant impact. Simulation of grain yield and particularly LAI could further be improved, and comparison against measured field data would be instrumental to succeed in doing so. Secondly, satellite-derived LAI products are affected by measurement and retrieval errors which introduce noise in the time series. Smoothing methods such as double logistics, splines, adaptive Savitzky-Golay filters^51^, or canopy structural dynamic models^52^ have successfully been applied to reconstruct temporal trajectories and improve the signal-to-noise ratio. LAI also correlates non-linearly with reflectance and tends to saturate over dense canopies (LAI values > 4)^53,54^. Error-adjustment methods have been proposed when ground measurements of LAI are available^55^. Recent empirical evidence converged inemphasising the importance of red-edge bands for operational estimation of biophysical parameters^56–58^ to bypass this saturation effect^57^ as well as to reduce some impacts of leaf angle distribution^58^. LAI estimates obtained from Sentinel-2, which carries three red-edge bands, are thus expected to improve in the near future. Finally, there might be a less than perfect agreement between the LAI values obtained from APSIM and those retrieved from space, even in the absence of noise or saturation. Indeed, satellites sense green LAI because the electromagnetic radiation reflected from the crop canopy is contributed by all the aerial plant organs^55^. Adjustment techniques might thus be required to improve the correspondence between these two LAI quantities. Despite these shortcomings, further developing approaches that calibrate empirical models with data generated from crop growth models is essential to reduce the burden of *in situ* yield measurement and to advance yield monitoring across the globe.

## Conclusions

The lack of suitable combinations of spatial and temporal resolutions of satellite image time series has long constrained large-area empirical yield estimation from space. Here, we sought to systematically evaluate how temporal resolution affects empirical relationships between wheat yields and descriptors of crop canopy dynamics as observed in leaf area time series and, in turn, to define their optimal conditions of use. Using the crop growth model APSIM as a data generator, we developed an *in silico* approach which allowed us to explore a wider range of G × E × M combinations than what observational data currently permit as well as to simulate the temporal resolutions of current and forthcoming satellites or satellite constellations.

We simulated wheat crops across Australia and regressed six types of metrics derived from the resulting time series of Leaf Area Index (LAI) against wheat yields. Empirical models solely based on LAI metrics captured between 30 to 80% of the wheat yield variability, the highest accuracy being achieved with advanced metrics (R^2^ > 75; Senescence fit and Fourier decomposition). This provides evidence that empirical metrics that truly harness the temporal dimension of LAI data and exhibit national relevance. Adding weather variables doubled the R^2^ values of models based on simple metrics (R^2^ > 0.55; Peak LAI and Early/Late windows) but had no significant improvement for those based on advanced metrics. This indicates that metrics intensively exploiting the temporal dimension already reflect most of the influence of weather on crop yield. Finally, simple metrics emerged as the best choice when dealing with sparse time series, *e*.*g*., 16 days, but were gradually outperformed by advanced metrics as the temporal resolution increased.

As we progress in a data-rich era, our findings support a general shift in the use of large-area empirical yield mapping towards the inclusion of metrics that truly harness the temporal dimension of leaf area data.

## Methods

### Wheat modelling across Australia

Australia is one of the top five wheat exporting countries in the world and accounts for 11% of global wheat trade during 2015^59^. It is estimated that 55% of Australian cropland is occupied by the current wheat area of *ca*. 14 Mha. Wheat yields in Australia have experienced substantial increase but evidence suggests that they have stalled at an average of 1.7 t ha^−1^ since 1990^60^. Wheat is sown around mid-May and is harvested from November to January^61^. The average field size exceeds 100 ha and irrigation is marginal.

**Figure 6:**
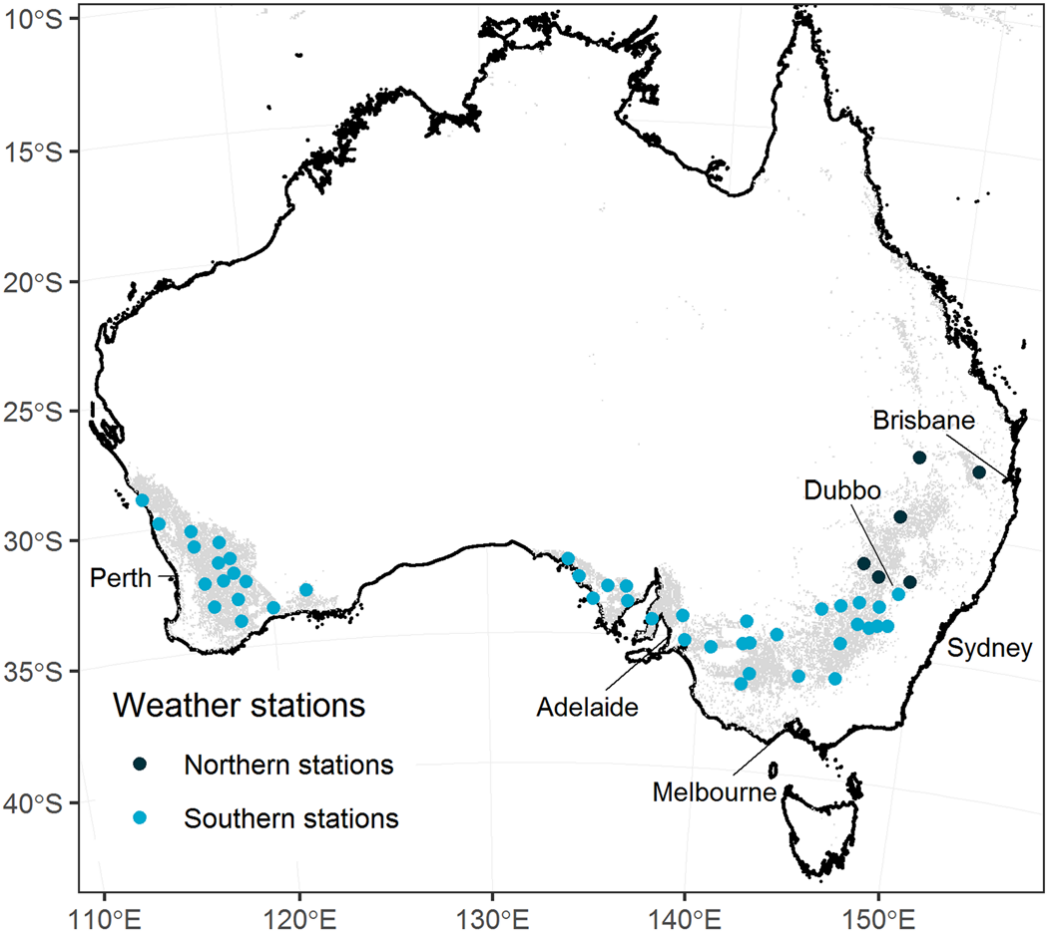
Location of the 50 high-quality weather stations. The area in grey indicates the cropland area as depicted in the Unified Cropland Layer^84^. Light blue dots indicate Southern stations whereas dark blue dots correspond to Northern stations.

We deployed the APSIM-Wheat model Version 7.8^19^ to grow continuous wheat from 1981 to 2015 at 50 high-quality weather stations representative of the Australian grain zone (Fig. 6). APSIM is a process-based model that simulates crop growth and development at a daily time-step in response to weather, soil water, soil nitrogen, and management practices. It calculates daily biomass accumulation using light interception and radiation use efficiency which is penalised under water and nitrogen stresses. Growth of leaf area is modelled daily using initial leaf area, leaf appearance rate and the relationship between plant leaf area and their processes are sensitive to daily temperatures. Grain yield is a function of grain number and grain weight. Grain number is determined pre-anthesis by stem weight and subject to reduction due to water stress during anthesis. Final weight per grain is determined by carbohydrate remobilisation, photosynthesis during grain filling, and the grain filling period which is accelerated by temperature and water stresses.

We used a state-of-the-art parameterisation of APSIM for the dominant soil types and nine management rules (see Hochman and Horan^62^ for more details). Similar model parameterisation at these locations explained ≥65% of the national and sub-national wheat yield variability^60,63^. Therefore, the simulation outputs were assumed to be reliable and no further validation was undertaken. The nine management scenarios were variants of standard simulation rules and covered a range of cropping practices (Tables 1 and 2). These included changes in the rate of nitrogen fertilisation (N-fertilisation), plant density (50, 75, 100, and 125 plants ha^−1^), sowing rule (Sow-1, -2, and -3), and fallow management. All sites in Queensland and northern New South Wales above latitude −32.24° were classed as northern sites and used the northern sowing rule, all other sites used the southern sowing rule. If the sowing criteria were not met during the sowing window, a crop was automatically sown on the 15^th^ of July. We considered five wheat cultivars spanning the range of Australian maturity types, namely: Bolac, Endure, Wyalkatchem, Derimut, and Correll. The parameterisation of these varieties was kept to their default values. Daily weather records of rainfall, maximum and minimum temperature (max and min T), and vapour pressure deficit (VPD) were sourced for the period of interest from the Australian Bureau of Meteorology^64^. Model runs from 1981 to 1999 were used to reach a credible soil water content and were thus discarded in further analysis. Simulations with a maximum LAI value < 1 were also discarded because they were likely associated with simulations of failed crops. The final data set had 7,712 entries that consisted of daily values of LAI, thermal time, phenological stage, and end-of-season yield.

**Table 1:** Management practices and the initial soil conditions of the standard simulation.

Parameters	Rules
*Sowing*	
Sowing rule	*Northern sites*
	Sow if rain ≥15 mm over 3 days and plant available water ≥30 mm from 26 April to 15 July
	*Southern sites*
	Sow if rain ≥15 mm over 3 days regardless of soil moisture from 26 April to 15 July
Sowing density	150 plants m^−2^
Sow spacing	250 mm
Sowing depth	30 mm
*Fertiliser*	
At sowing	Add 100 kgN ha^−1^ minus soil nitrate N in the top 60 cm of soil on April 25
In-season	Check top 60 cm soil daily, if NO_3_ <80 kg ha^−1^, plant available water ≥30 mm and Zadok’s growth stage ≥10 and ≤49 then add 70 kgN ha^−1^ (maximum 1 application)
*Soil*	
Initial soil water	10% of plant available water capacity
Initial soil NO_3_	25 kg ha^−1^ for each metre depth of soil
Initial soil NH_4_	5 kg ha^−1^ for each metre depth of soil
Surface organic matter	100 kg ha^−1^ with a Carbon:Nitrogen ratio of 80

**Table 2:** Changes made to the standard simulation for the nine treatments.

Treatment Code	Rules
N-fertilisation	Apply different rates of N depending on whether Yw is low, medium or high:
	If Yw ≤3.2 t ha^−1^, apply 22.5 kgN ha^−1^ at sowing only.
	If Yw >3.2 t ha^−1^ and Yw ≤4.4 t ha^−1^, apply 45 kgN ha^−1^ at sowing only.
	If Yw >4.4 t ha^−1^, apply 67.5 kg N ha^−1^ at sowing only.
	Soil N and surface organic matter are not reset
	Initial soil N 124 kgN ha^−1^ distributed through layers of the profile
Plants 50	Sowing density changed to 50 plants m^−2^
Plants 75	Sowing density changed to 75 plants m^−2^
Plants 100	Sowing density changed to 100 plants m^−2^
Plants 125	Sowing density changed to 125 plants m^−2^
Sow-1	*Northern sites*
	Sow if rain ≥25 mm over 3 days and PAW ≥30 mm from 26 April- to 15 July.
	*Southern sites*
	Sow if rain ≥25 mm over 3 days regardless of soil moisture from 26 April to 15 July.
Sow-2	*Northern sites*
	Sow 2 weeks after rain ≥15 mm over 3 days and PAW ≥30 mm from 26 April to 15 July.
	*Southern sites*
	Sow 2 weeks after rain ≥15 mm over 3 days regardless of soil moisture from 26 April to 15 July.
	If sowing criterion is not met by 15 July, sow 2 weeks after 15 July.
Sow-3	Sow using highest yielding sowing date from analysis of crops sown every 7 days from 5 April to 21 June using highest yielding cultivar.
Fallow	To simulate the effect of weeds during the fallow, plant available water was reduced by up to 30 mm on 25 April by removing 70% of the plant available water from each layer starting from the top layer

Yw: water-limited yield potential; PAW: plant available water.

**Figure 7:**
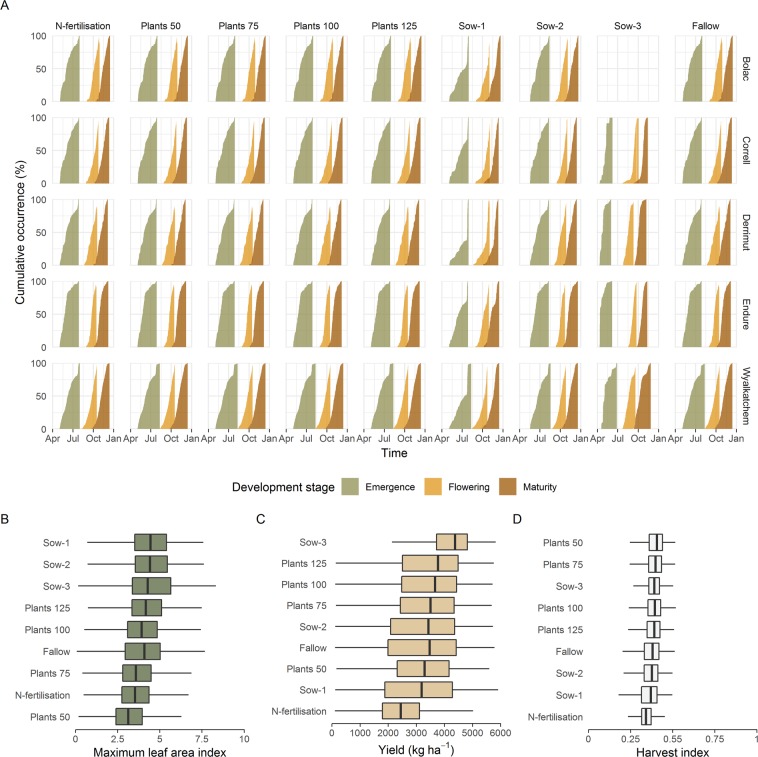
Output of the crop growth simulations: (**A**) cumulative occurrence of phenological stages per treatment and variety, distributions of (**B**) Maximum leaf area indices, (**C**) yields, and (**D**) harvest indices per treatment.

We summarised the main characteristics of the simulation outputs in Fig. 7. Emergence started as early as April 4^th^ (Sow-3) and finished as late as August 18^th^ (Sow-2). Flowering ranged from July 22^nd^ (Sow-3) to November 17^th^ (Sow-2), which covered reported flowering periods^37,65–67^. Maturity occurred from September 9^th^ (Sow-3) to December 19^th^ (Sow-1), with strong differences across treatments. Under the Sow-1 strategy, wheat was sown before the cutoff date of July 15^th^ in *ca*. 50% of the cases. Note that Bolac was never the highest yielding variety so no Sow-3 simulation was available for that variety. Maximum LAI values averaged 3.93 across simulations with maximum values up to 8.34 (Sow-3). Simulated yields averaged 3247 kg ha^−1^ with a range of 105 kg ha^−1^ to 5,824 kg ha^−1^. Harvest indices (the ratio of grain yield and biomass) averaged 0.375 across simulations, spanning from 0.178 (Sow-1) to 0.518 (Plants 100). Further, yields and harvest indices were within the range of values reported in an exhaustive search of the literature for dryland wheat in Australia^68^. Therefore, we concluded that our simulations provided realistic scenarios of dryland wheat growth across the Australian wheat belt.

### Predicting grain yields with empirical models

The empirical yield prediction model followed the following form:

1 $$Y={\beta }_{0}+{\beta }_{1}{\bf{X}}+{\beta }_{2}{\bf{W}}$$

where **X** is a vector of LAI metrics derived from simulated LAI time series, **W** is a vector of weather attributes over the season, and *β*_0_, *β*_1_, and *β*_2_ are the associated coefficients. First, we restricted the empirical model to LAI metrics (*Y* = *β*_0_ + *β*_1_**X**) and assessed its performance for three time scales (calendar time, thermal time, and phenological time). Secondly, the added-value of weather variables (**W**) for yield prediction was evaluated and the importance of weather variables was quantified by partitioning the coefficient of determintation. Thirdly, we investigated the loss in accuracy due to reduced observation frequencies. Finally, we mapped optimal metrics for three temporal revisit frequencies (5, 10 and 16 days) across the Australian wheat area.

#### Yield prediction with LAI metrics

To quantify the yield variability explained by LAI metrics, we extracted six groups of metrics from the simulated LAI time series provided by APSIM (Table 3).

**Table 3:** Description of the LAI metrics derived by the six methods.

Method	Predictors	Description	Selected references
1. Peak LAI	*m*_*all*_	Maximum LAI value	^9^
2. Early/Late Windows	*m*_*pre*_	Maximum LAI value in a pre-anthesis time window	^18^
	*m*_*post*_	Maximum LAI value in a post-anthesis time window	
3. Integral	*I*_*all*_	Area under the seasonal LAI curve	^8^
4. Partial Integral	*I*_*post*_	Area under the decreasing part of the LAI curve	^39^
5. Senescence Fit	*m*_*all*_	Maximum LAI value	^85^
	*k*	Senescence rate	
	*p*	Position of the inflection point in the decreasing part of the curve	
6. Fourier Decomposition	*a*_0_	Mean value of the function over one period	This study
	*a*_1–2_	Amplitude of the two first harmonics	
	*ϕ*_1–2_	Phase of the two first harmonics	

The first approach (Peak LAI) identifies the maximum LAI value from the time series as it corresponds to the onset of the reproductive stage which is a critical period for the determination of wheat yield^69^. Empirical evidence has also shown that the best single-date correlation between wheat yield and LAI occurs at the time of highest LAI which concurs with the transition from the vegetative stage to grain filling^9,36^.

In the second method (Early/Late Windows), maximum LAI values observed during two windows, one early (day of year 203–day of year 253) and the other late in the season (day of year 274–day of year 314) were derived^18^.

Integration of seasonal LAI profiles was also examined a third and fourth method for feature extraction. The integration of satellite observations over time was shown to represent the intensity and the duration of the photosynthetic activity of the crop throughout the growing cycle well and, as a result, it was highly correlated with the actual yield^8,70,71^. The definition of the integration interval is critical and previous work recommended to start from the beginning of nutrient substance accumulation in storage organs^39^, which corresponds to flowering in wheat, rather than from the beginning of the crop cycle. We compared these two integration approaches and computed the area under the curve for the entire LAI profile (Integral) and from peak LAI to harvest (Partial Integral).

The fifth approach (Logistic Fit) estimated wheat yield from three parameters describing the crop senescence^72,73^. These were obtained by fitting a modified logistic model to the LAI time series^74^:

2 $${\rm{LAI}}\,(t)=\frac{{m}_{all}}{1+\exp \,(\,-\,k[t-p])}$$

where *m*_*all*_ refers to the maximum value of LAI, *p* is the position of the inflection point in the decreasing part of the LAI curve, *k* is the relative senescence rate, and *t* is input time.

Finally, we used Fourier Decompositions which is an approach known to capture the temporal dynamics while reducing the dimension and the noise^75^. Fourier Decomposition transforms an input signal from the time domain into the frequency domain. In a closed interval [0; *N*], this approach assumes that the signal *f*(*t*) can be decomposed into a series of sine-waves with increasing frequencies^76^:

3 $$f(t)=\frac{{a}_{0}}{2}+\mathop{\sum }\limits_{i=1}^{i=N/2}{a}_{r}\,\cos \,(\frac{2\pi it}{N}-{b}_{i})$$

The result of a discrete Fourier transform is a complex number with a real (*a*) and an imaginary (*b*) part that can be converted to polar form. Then, each harmonic wave *i* can be defined by a phase and an amplitude^77^:

4 $${a}_{i}=\sqrt{{a}_{i}^{2}+{b}_{i}^{2}}$$

5 $${\varphi }_{i}=\arctan \frac{{b}_{i}}{{a}_{i}}$$

Together with the additive term (*a*_0_), the harmonic components can together reconstruct the initial signal. By discarding higher order harmonics, it is possible to retrieve lower noise signal. In this study, we kept the additive term and the first two harmonics as predictors of yield.

#### Three time scales

Measuring time in calendar days has been the dominant approach in remote sensing because it matches the acquisition dates of the satellite images. However, this approach might be limited when dealing with large G × E × M variations, *e*.*g*., for estimating yield at a national scale. There is a considerable advantage in describing crop development based on thermal time units as the duration in thermal time required to reach a certain ontogenetic phase is relatively constant, while that in calendar days may considerably vary^78^. Relying on phenological stages is a further refinement that accounts for vernalisation and/or photoperiod requirements which affect the rate of crop development.

All LAI metrics were derived for these three time scales: calendar time, thermal time, and phenological stages. Thermal time was computed following Zheng *et al*.^79^ and the phenological stages were described using Zadok’s decimal scale^80^ as simulated by APSIM. The Zadok’s growth scale is based on ten principal cereal growth stages from germination to ripening, each of these is divided into ten secondary stages, extending the scale from 00 to 99.

#### Model evaluation

Empirical models were calibrated using 50% of the data set (n = 3,845) and validated with the remaining 50% (n = 3,867). Note that, to avoid any bias, the split between the calibration and validation sets was done to guarantee that all simulations relative to a station-year would either belong to the calibration or validation set. The performance of the models was quantified using the Root Mean Square Error (RMSE) and the coefficient of determination (R^2^). The RMSE gives the weighted variations in error (residual) between the predicted and observed yields while the R^2^ expresses the percentage of variance explained by the model.

#### Contribution of weather metrics

First, the accuracy of a yield model only based on weather features (*Y* = *β*_0_ + *β*_2_**W**) was quantified. To that aim, we extracted 14 weather variables by averaging daily observations (VPD, min and max T) and summing daily observations (P, VPD, min and max T) before and after the peak of LAI. We then evaluated the merit of adding meteorological features to the empirical model in order to boost the prediction accuracy. All models were recalibrated to consider the weather variables, and the net effect on the R^2^ and the RMSE was measured. To identify the most relevant variables, relative importance metrics for linear models were computed by partitioning the coefficient of determination^81,82^.

### Robustness to reduced temporal frequencies

So far, all models were calibrated on LAI metrics derived from gap-less daily time series. As these are gap-less daily time series, they set the upper limit in terms of attainable accuracy, their performances might significantly change with sparser time series resulting from coarser temporal resolutions or missing values due to cloud/cloud shadow contamination. To provide insights on their generalisation potential, we applied the previously calibrated models to LAI metrics extracted from time series with lower temporal resolution, accounting for cloud conditions.

Daily, 5-day, 10-day, and 16-day LAI time series were created to simulate the revisit cycles of the Dove constellation, the Sentinel-2A or/and -2B, and Landsat-8. The remaining LAI values were further removed according to their corresponding daily cloud probability. Monthly mean cloud frequencies were sourced from Wilson and Jetz^83^. This data set integrates 15 years of twice-daily remotely sensed cloud observations at 1-km resolution. We applied a linear interpolation to generate daily cloud probability assuming the monthly average was representative of the 15^th^ of each month. To avoid artifacts, values for December and January were duplicated at the end and the start of the time series, respectively. Therefore, daily cloud probabilities were interpolated based on an input time series of 14 values and the first 16 (December 15^th^–December 31^st^) and last 15 values (January 1^st^–January 15^th^) were discarded. Missing values in the LAI time series were then linearly interpolated prior to yield estimation. A Monte Carlo approach was used and this process was repeated ten times. The impact on the prediction was measured using the average RMSE across the ten realisations. As an additional evaluation criterion, the number of times the metrics computation failed due to a lack of input data was computed.

Finally, the optimal metrics were identified for each temporal resolution. These were then interpolated to the Australian wheat production area at a 1-km^2^ resolution based on a nearest-neighbour search. In other words, pixels were attributed to the best-performing metrics of the station they were the most similar to in terms of cloud frequency. Similarity between cloud patterns was measured with the Euclidean distance.

## Data Availability

Thee datasets generated and/or analysed during the current study are available from the corresponding author on reasonable request.
